# Bidirectional and Stretchable Piezoresistive Sensors Enabled by Multimaterial 3D Printing of Carbon Nanotube/Thermoplastic Polyurethane Nanocomposites

**DOI:** 10.3390/polym11010011

**Published:** 2018-12-21

**Authors:** Josef F. Christ, Nahal Aliheidari, Petra Pötschke, Amir Ameli

**Affiliations:** 1 Advanced Composites Laboratory, School of Mechanical and Materials Engineering, Washington State University, 2710 Crimson Way, Richland, WA 99354, USA; josef.christ@wsu.edu (J.F.C.); nahal.aliheidari@wsu.edu (N.A.); 2 Department of Functional Nanocomposites and Blends, Leibniz Institute of Polymer Research Dresden, Hohe Straße 6, Dresden D-01069, Germany; poe@ipfdd.de

**Keywords:** carbon nanotubes, functional nanocomposites, additive manufacturing, 3D printing, fused filament fabrication, strain sensing, piezoresistivity

## Abstract

Fabricating complex sensor platforms is still a challenge because conventional sensors are discrete, directional, and often not integrated within the system at the material level. Here, we report a facile method to fabricate bidirectional strain sensors through the integration of multiwalled carbon nanotubes (MWCNT) and multimaterial additive manufacturing. Thermoplastic polyurethane (TPU)/MWCNT filaments were first made using a two-step extrusion process. TPU as the platform and TPU/MWCNT as the conducting traces were then 3D printed in tandem using multimaterial fused filament fabrication to generate uniaxial and biaxial sensors with several conductive pattern designs. The sensors were subjected to a series of cyclic strain loads. The results revealed excellent piezoresistive responses with cyclic repeatability in both the axial and transverse directions and in response to strains as high as 50%. It was shown that the directional sensitivity could be tailored by the type of pattern design. A wearable glove, with built-in sensors, capable of measuring finger flexure was also successfully demonstrated where the sensors are an integral part of the system. These sensors have potential applications in wearable electronics, soft robotics, and prosthetics, where complex design, multi-directionality, embedding, and customizability are demanded.

## 1. Introduction

The wearable electronics and technology market is estimated to grow to $150 Billion by 2026 [1]. There has been significant research within the field of wearable electronics for developing smart sensors and functional textiles [2,3,4,5,6,7,8]. Soft robotics is another area that demands novel sensing systems [2,9]. As the desire for advanced wearable electronics grows and the soft robotics industry advances, developing novel sensing materials and systems becomes increasingly important. There have been many attempts at developing new materials [10,11,12,13] and characterizing piezoresistive behavior [14,15,16,17,18,19] for strain sensing applications. In the field of strain sensing, there is a great demand for flexible sensors, due to their high potential in various applications, especially wearable electronics and soft robotics, and significant research attempts have been made towards developing highly flexible sensors capable of measuring large strains [4,5,9,20,21].

Currently, one of the major challenges in the strain sensing technologies is their fabrication processes. Some of the current methods used for fabricating sensors include photolithography [22,23,24], vacuum filter deposition [25,26], evaporative deposition [8,20,24,27,28,29,30,31], lamination [24,30], coating [32], solution casting [33], and inkjet printing [34]. Unfortunately, many of these methods are time-consuming, complex, and with limited design flexibility, not allowing for easy sensor scalability and/or geometrical designs capable of multidirectional sensing. Specifically, solution casting, which has been traditionally used, suffers from complexity of fabrication, scalability, and sometimes unhealthy solvents. There still lacks a strong method for fabricating complex sensor platforms. This research aimed to bridge this schism between the design of strain sensing platforms and the fabrication through the implementation of fused filament fabrication (FFF) additive manufacturing.

Additive manufacturing has seen a rapid growth in adoption within academia and industry with research being focused in characterizing and demonstrating 3D printing as a powerful tool for fabrication [35,36,37,38,39,40,41,42,43]. Recently, there has been some research work done towards the implementation of 3D printing methods in fabricating strain sensors; these works include using conductive inks printed within flexible elastomers [21], combining FFF printed substrates with conductive adhesives [44], conductive solvent-cast ink printing [45], and printing polylactic acid (PLA)/carbon nanotube composite filaments using FFF for rigid and low strain sensors [46]. However, to the best of our knowledge, the ability to fabricate multidirectional, embedded, and patterned sensors of high strain using FFF 3D printing has not yet been demonstrated.

In FFF additive manufacturing, the feedstock, typically a thermoplastic-based polymer, is fed into a heated nozzle where it is then extruded as a continuous bead of molten plastic. The nozzle is maneuvered by a computer numerical control (CNC) machine, which directs the path of the nozzle to pattern out the part as a series of discrete layers, building the part from the bottom, moving upward [38,47,48,49]. An advantage of FFF technology is the ability to print multiple materials within a single print. The multimaterial printing capability of FFF has been an underutilized tool for fabricating complex and hybrid mesostructures, including sensor designs, as it can allow for the direct integration of sensing materials within structural systems. Typically, sensors are added to structures or systems after-the-fact, increasing system complexity or cost. By integrating sensing platforms directly within the structure, the desired measurement locations can be more readily tuned for better data acquisition. Multidirectional sensors can be used for the simultaneous measurement of strain in various directions. Special patterns of sensing elements can be designed for optimized sensitivities or other design merits. The multi-directionality, embedding, and patterning can be potentially achieved collectively or selectively using FFF method. The multimaterial printing capability of FFF method coupled with its CNC-controlled movements can potentially result in the deposition of conductive paths in any location and pattern (2-D and 3-D) while embedded within an overall insulative matrix.

Conductive polymer composites (CPCs) that contain conductive fillers, specifically carbon-based fillers, have attracted significant interest, due to the combination of properties that they offer. They have functional applications as semi-conductive and conductive materials in sensors, super-capacitors, and electromagnetic shields [50,51,52,53,54]. CPCs have already demonstrated significant functional uses within the field of strain sensing. A few of the most commonly used conductive fillers within CPCs are graphene [9,24,26,55], carbon black [19,46], and single- and multiwalled carbon nanotubes [22,23,32,46,56,57,58]. Within this research, a conductive TPU/MWCNT nanocomposite was chosen due to the highly elastic nature of TPU [59] as well as its good thermoplastic performance for FFF printing. MWCNT material was chosen as the filler due to its superior conductive properties when composited within thermoplastics, even at very low loading contents [12,14,50]. MWCNT composites also demonstrate the very good piezoresistive behavior [12,17,30,31,60] needed for strain sensing applications. Recently, it has been reported that 3D-printed TPU/MWCNT exhibits good strain [61] and force [62] sensing behaviors. However, the integration of the nanocomposite as a sensing element in a multimaterial system is yet to be demonstrated. 

In this work, the fabrication and characterization of bidirectional strain sensors obtained through the tandem printing of bi-materials, i.e., conductive TPU/MWCNT and insulative TPU is reported. The piezoresistive behavior of uniaxial and biaxial sensors with several patterns was investigated under cyclic loadings with strains as high as 50%. The impact of the designed patterns on the sensing was analyzed. The microstructure and the interlayer bonding are also discussed. Further, a wearable glove with integrated sensing components to measure finger flexure is demonstrated as a potential application.

## 2. Materials and Methods

### 2.1. Materials

The materials used in this research include Elastollan^®^ 1185A TPU (Density: 1.12 g/cm^3^, Shore hardness: 85 A, Elastogran GmbH, Lemförde, Germany) and MWCNTs (Nanocyl^®^ NC7000™, Nanocyl S.A., Sambreville, Belgium, 90% purity, average diameter: 9.5 nm, average length: 1.5 µm, volume resistivity: 10^−4^ Ω·cm). Commercial NinjaFlex (Shore hardness: 85 A, TPU composition, NinjaTek, Manheim PA, USA) and PLA Ingeo 2003D (Density: 1.25 g/cm^3^, NatureWorks LLC, Minnetonka, MN, USA) FFF printer filaments were also used for demonstrating commercial integration capabilities.

### 2.2. Filament Fabrication

TPU filament containing 3 wt % MWCNT was produced by diluting a 5 wt % masterbatch of TPU/MWCNT (made by Berstorff ZE 25 extruder, screw length-to-diameter ratio: 36 L/D, melt temperature: 221–229 °C, rotational screw speed: 300 rpm, and throughput: 10 kg/h; carbon nanotubes were dosed into a side feeder attached at 14D) with pure TPU Elastollan^®^ 1185A through a 16 mm twin-screw extruder type LTE with L/D ratio of 16/40 (LabTech Engineering Company LTD., Muang, Samutprakarn, Thailand). Pure Elastollan^®^ 1185A TPU filament was produced by extruding TPU pellets through the same 16 mm twin-screw extruder.

### 2.3. Sensor Fabrication

**Figure 1:**
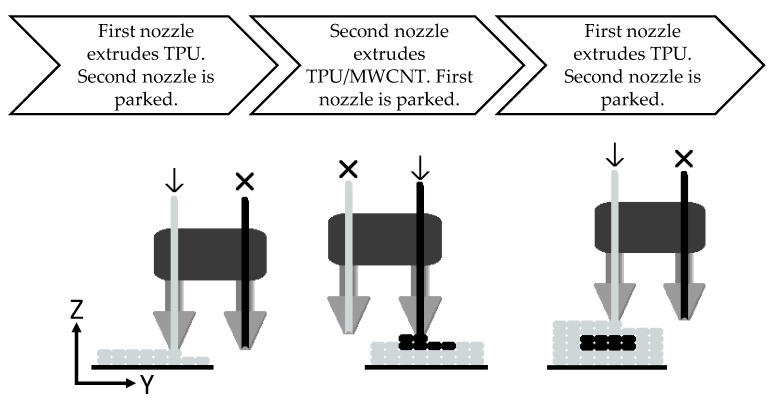
Schematic illustration of the sequences in multimaterial printing process through FFF. Nozzles move in the x-direction, normal to the page.

**Figure 2:**
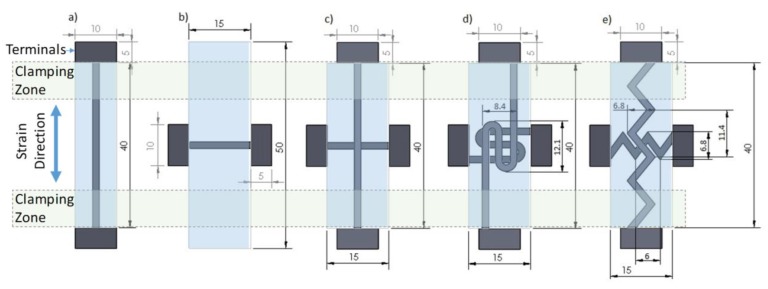
CAD drawings for: (**a**) linear axial; (**b**) linear transverse; (**c**) linear biaxial; (**d**) switchback biaxial; and (**e**) sawtooth biaxial. All dimensions are in mm. Within each design, the black and light blue regions are TPU/MWCNT and pure TPU, respectively. The electrical connection terminals, clamping zone, and strain direction are also identified. The sample thickness in (**a**,**b**) is 1.0 mm and in (**c**–**e**) is 1.2 mm. The clamping zone size is 7.5 mm on each side, leaving a free length of 25 mm between the clamps.

For sample preparation, a Makerbot Replicator 2x experimental printer (MakerBot Industries, LLC, Brooklyn, NY, USA) was used. The layer resolution, positioning precision in the build plane (X-Y), and the positioning precision in the build direction (Z) of the utilized printer are 100, 11, and 2.5 µm, respectively, according to the printer manual. Table 1 shows the optimized printing parameters used in the fabrication of all the samples. The 3D printer offers two independent extrusion nozzles, which allow for the use of two independent materials during printing. Dual extrusion functioned as schematically shown in Figure 1: one nozzle deposited the pure TPU filament, while the second deposited the TPU/MWCNT filament. All model designs were prepared using SolidWorks (Dassault Systèmes SOLIDWORKS Corp., Waltham, MA, USA). Figure 2 shows the CAD drawings of the sensor designs and the overall sensor dimensions noting that the single axis sensors are 1.0 mm thick and the biaxial sensors are 1.2 mm thick. The conductive paths were dimensioned at 1.6 mm wide by 0.4 mm thick.

**Figure 3:**
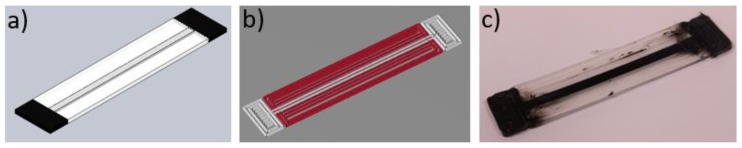
Design-to-fabrication process: (**a**) generating CAD model; (**b**) rendering code for 3D printing; and (**c**) resulting physical sensor.

The software used to convert the 3D models into printable formats as well as control the 3D printer was Makerware Desktop 3D printing software (MakerBot Industries, LLC, New York city, NY, USA). This software converts the CAD model into a series of discrete coordinate positions, which controls the printer’s extruder position. Figure 3 provides a visualization of the sensor during the design-to-fabrication process.

**Figure 4:**
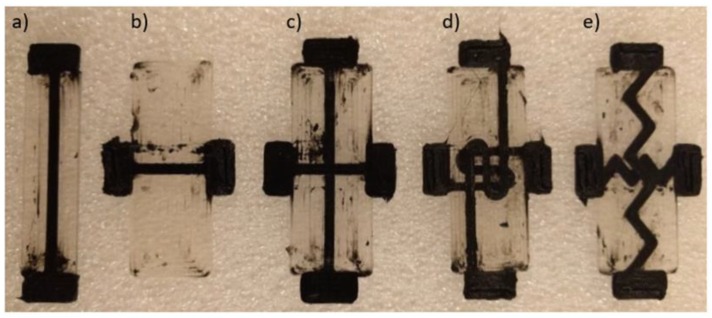
Photos of the actual printed sensors: (**a**) linear axial; (**b**) linear transverse; (**c**) linear biaxial; (**d**) switchback biaxial; and (**e**) sawtooth biaxial. The dimensions are dictated by the CAD models of Figure 2.

Figure 4 shows the resultant printed sensor designs. Each sensor design was fabricated in a single run of the printer with no further post-processing. The biaxial designs have two sets of conductive traces, which lie on different planes demonstrating the capability to print multi-layer, multidirectional sensor designs without increasing the number of steps required during fabrication. It is noted that, due to the dual nozzle extrusion, while the TPU/MWCNT nozzle is parked, it is still moving along with the extruder carriage. Any dark spots or blurred regions within the pure TPU, as shown in Figure 3 and Figure 4, are results of minor residual TPU/MWCNT being smeared from the nozzle tip of the parked extruder on the surface of the TPU rasters during the printing process. Therefore, they do not affect the sensor performance, which is governed by the conductive traces. They are more of aesthetic imperfections and can be avoided or decreased by optimizing the nozzle trajectory.

### 2.4. Characterization

For microstructural observations, scanning electron microscopy (SEM) was conducted on cryo-fractured surfaces to illuminate the layered structure and layer adhesion (at lower magnification) as well as nanotube dispersion and orientation (at higher magnification). Samples were immersed in liquid nitrogen and broken perpendicular and parallel to the printing direction. The samples were sputtered with ca. 3 nm platinum and imaged using A Carl Zeiss Ultra plus microscope (Carl Zeiss Microscopy GmbH, Jena, Germany) by applying the SE2 detector. 

All mechanical loading was carried out on a custom tensile testing machine at a 5 mm/s loading and 10 mm/s unloading rates with about 5 s holding time after each loading/unloading cycle, unless otherwise noted. A two-point resistance measurement method was used for the in-situ resistance measurement during strain loadings. This was achieved by applying an input voltage, *V*_in_, of 10 volts load to the sensor connected in series to a known resistance, *R*_2_. The voltage on the sensor was applied using the contact pads, as identified by terminals in Figure 2 for various sensor cases. The output voltage, *V*_out_, the voltage across the sensor only, was measured and recorded using a data acquisition system. Through this standard voltage divider circuit, the resistance of the sensor, *R*_1_, was then found using:

(1) $$R_{1}=R_{2}\left(\frac{V_{\mathrm{in}}}{V_{\mathrm{out}}}-1\right)$$

It is noted that, in the voltage divider, *R*_1_ and *R*_2_ are connected in series and *V*_in_ is applied to both while *V*_out_ is measured on only the sensor. To minimize the surface resistance, the metal contact pins were heat fused onto the printed contacts to provide a reliable connection.

## 3. Results and Discussion

### 3.1. Microstructure

**Figure 5:**
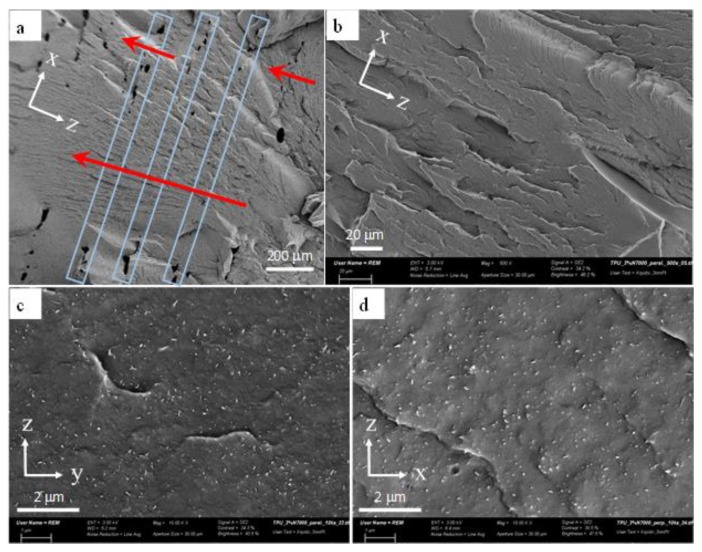
SEM micrographs of FFF 3D printed TPU/3 wt % MWCNT samples. x is the layer deposition direction (i.e., the direction of nozzle movement), y is the width direction and z is the height direction where layer stacking takes place. (**a**) x-z view of the interlayer bonds. The blue rectangles indicate the interlayer areas and the red arrows point the regions where the failure flows across the interlayer regions. (**b**) A view of x-z plane showing an intralayer region with no signs of large agglomerates. (**c**,**d**) The MWCNT dispersion and orientation in the planes normal (z-y) and parallel (x-z) to the layer deposition direction (x axis), respectively.

An important factor that influences the mechanical and physical properties of printed nanocomposites is the quality of layer-to-layer bonding during layer deposition. Figure 5a gives a SEM micrograph where the interlayer and intralayer regions of TPU/3 wt % MWCNT can be examined. It is seen that the layers are orderly stacked in the height direction (z direction) with a relatively uniform height of ~200 μm. In printed rigid plastics such as acrylonitrile butadiene styrene or polylactide, there are usually continuous linear voids along the layer deposition direction at the vicinity of the interlayer regions. Such regions of TPU/3 wt % MWCNT are identified by blue rectangles in Figure 5a, where no continuous linear voids are observed and the failure partially flows across the interlayer regions, as pointed by the red arrows. This mesostructure characteristic indicates a relatively wide-area interlayer bond, which contributes to having less volume of voids and enhanced mechanical and physical properties.

To obtain enhanced and reliable electrical conductivity and mechanical properties in nanocomposites, it is important to have a consistent dispersion of MWCNTs within the polymer matrix. Further, for effective FFF printing process, it is critical that the MWCNT agglomerates are minimized in size and number within the TPU matrix, as agglomerates can cause nozzle clogging and impede the printing process. As shown in Figure 5b, there is no indication of micro-sized agglomerates within TPU/3 wt % MWCNT layers on the surface of a fractured layer. Figure 5c,d also shows the SEM micrographs, taken on the planes normal and parallel to the layer deposition axis. The MWCNTs have a relatively uniform distribution with an excellent state of dispersion, as confirmed by the existence of many individual nanotubes. Short and individual striation marks on the surfaces might be a consequence of remaining carbon nanotube bundles. It is also interesting to note that overall no significant difference was observed in the alignment state of MWCNTs in the two different planes. In other words, the nanotubes were not significantly aligned in the extrusion direction (x axis). This agrees well with the fact that the in-layer and through-layer conductivities of printed TPU/MWCNT nanocomposites were reported to be very similar in magnitude [61], indicating a relatively random alignment of carbon nanotubes, instead of machine direction alignment.

### 3.2. Mechanical Behavior

**Figure 6:**
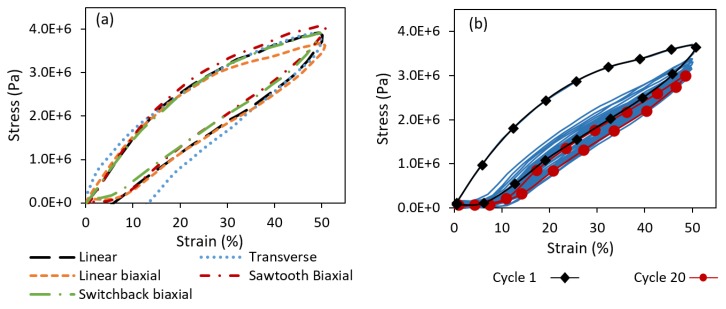
(**a**) Stress–strain response of all sensor types for loading and unloading with a maximum strain of 50%; and (**b**) cyclic stress–strain response for linear biaxial sensor at 20 cycles.

Figure 6a shows the stress–strain behavior of the five sensor designs over a single loading unloading cycle of 50% maximum strain. Figure 6b also shows the cyclic behavior of the linear biaxial sensor under a series of loading and unloading. Other sensor types showed similar cyclic behavior. As seen in Figure 6a, there is a minimal difference between the stress–strain behaviors of all five sensor types. This is because the majority of the sensor material is composed of pure TPU as the platform. One noticeable behavioral difference in Figure 6a is in the unloading of the transverse sensor, which exhibited a higher degree of strain softening effect. The strain softening effect is reflected by the magnitude of residual strain once the stress approaches zero at the end of the unloading step. Strain softening has been demonstrated as a common phenomenon, which occurs in filled elastomeric materials [63,64]. Strain softening is often associated with the rearrangement and breaking down of the polymer chains due to the applied strain. Higher degree of strain softening in the transverse sensors can be attributed to more damage formation at the interlayer regions, as this is the only sensor in which the interlayer bonds experience normal stresses. All other sensor types carried the applied load along the printed layers. A different state of molecular entanglement and inter-diffusion is expected at the interlayer region, compared to those in the bulk polymer and may thus result in a different strain softening response. As seen in Figure 6b, strain softening was mostly observed within the first few cycles and all the sensors demonstrated a very rapid (after ~4 cycles) approach to an equilibrium curve, demonstrating a strong potential for the use in cyclic loading scenarios. As shown in Figure 6b, another point of interest is the vertical downward shift on the curve with progressing cycles. This has been referred to as the Mullins effect or stress softening, commonly seen in filled rubbery materials [63]. It is demonstrated by a drop in the material stress and a stress hysteresis. The Mullins effect was also saturated within the first few cycles, indicating that the sensors could be load-treated before actual application to minimize the strain softening and Mullins effect.

### 3.3. Piezoresistive Behavior

**Figure 7:**
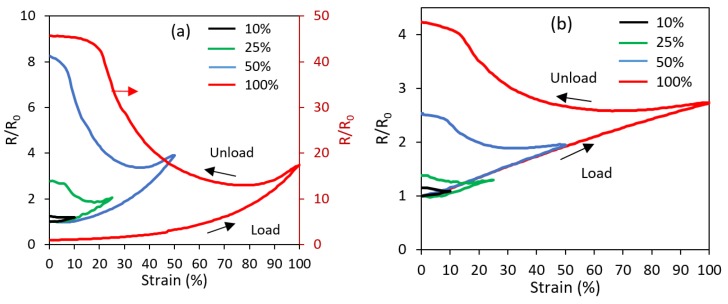
Relative resistance change (R/R_0_) during single loading-unloading cycle for (**a**) linear axial and (**b**) linear transverse sensors tested under various maximum strains ranging from 10% to 100%. The R/R_0_ response of 100% maximum strain loading in (**a**) is plotted against the secondary ordinate.

The acting mechanism for the piezoresistive response within the printed sensors is the change in the conductive network structure within the printed trace of TPU/3 wt % MWCNT. The piezoresistive response is often attributed to a mechanical deformation, reorientation, and dissociation of the conductive nanotube network within the polymer [14,65]. This network evolution increases or decreases the electron tunneling gaps, which in turn affects the overall resistance of the material [66]. Figure 7a,b shows the effect of different strain loadings on the relative resistance for the linear axial (Figure 4a) and linear transverse (Figure 4b) sensors, respectively. In the relative resistance of R/R_0_, R is the actual resistance at the time of measurement and R_0_ is the initial resistance before any straining. For the axial sensor (in which the strains are parallel the layer deposition direction), the resistance response tends toward an exponential behavior with strain, whereas, for the transverse sensor (in which the strains are normal to the layer deposition direction), the resistance behavior is far more linear. Furthermore, the resistance change of the linear sensor is markedly higher than what is demonstrated by the transverse sensor.

The difference in the magnitude of the resistance response between the axial and transverse sensors can be attributed to three factors: (a) the actual strains that the embedded TPU/MWCNT traces experience within the axial and transverse sensors; (b) the direction of the resistance measurement with respect to the alignment direction of nanotubes; and (c) the Poisson’s effect induced during straining of the sensors. Similar to the micromechanical analysis of a lamina in the mechanics of laminated materials [67], in the axial sensors, the actual strain of the conductive trace was the same as the external applied strain, enforced by the continuity and compatibility of the composite (strips are loaded in parallel). In the case of the transverse sensor, the stresses transferred from the TPU platform to the conductive traces remained unchanged, enforced by the static equilibrium (strips are loaded in series). Since the conductive trace of TPU/MWCNT had a higher modulus of elasticity, compared to the TPU matrix (measured to be ~2.5 times), the actual strain of the transverse conductive trace was thus lower, resulting in a smaller change in resistance. Therefore, it is interesting to note that, by tuning the modulus ratio of the platform polymer and the conductive trace, the transverse sensitivity can be manipulated. Furthermore, when a carbon nanotube composite is stretched by axial loading, the nanotubes are aligned in the stretching (axial) direction. Therefore, the resistivity was measured parallel (Figure 4a) and normal (Figure 4b) to the alignment direction of MWCNTs in the case of axial and transverse sensors, respectively. It is known that the resistance is direction dependent in aligned nanocomposites [68,69]. Using Monte Carlo simulation, Chang et al. showed that, upon the straining of a percolative system, the rate of resistance change (or percolation threshold change) with strain is different in the axial and transverse directions [70]. Moreover, once an object is stretched in one direction, there will be a compressive strain in the directions normal to the stretching direction, due to Poisson’s effect. Based on the piezoresistivity theory, dimensional change (Δ*R*_d_) and intrinsic piezoresistive effect contribute to the overall resistance change of a sensor under strain and the dimensional change is related to Poisson’s ratio, ν, by Δ*R*_d_ = 1 + 2ν [71,72]. Due to different stretching directions under different geometrical confinements of the embedded TPU/MWCNT traces within the axial and transverse sensors, the Poisson’s effect induced strain and resistance change will be different. This provides another credence to the lower resistance change values found in the transverse direction over the axial direction.

**Figure 8:**
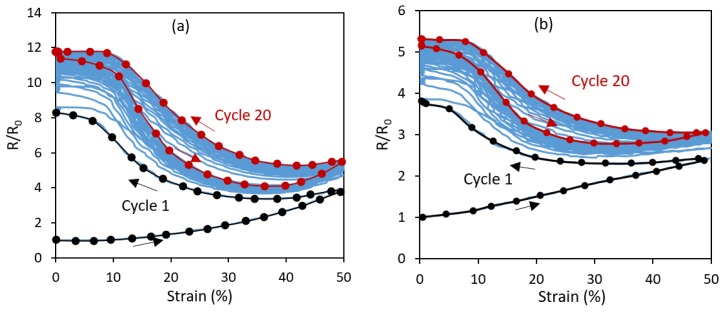
Relative resistance change (R/R_0_) over 20 loading-unloading cycles at 50% maximum strain for: (**a**) the linear axial sensor; and (**b**) the linear transverse sensor.

Another phenomenon shown in Figure 7a,b is the permanent increase of the sensor’s resistance upon unloading during the first cycle. This change became more pronounced at larger strain magnitudes. This is likely due to the non-recoverable slipping and disconnections within the MWCNT network. However, this non-recoverable response diminishes quickly with subsequent strain loadings. Figure 8a,b shows the cyclic loading and unloading at 50% maximum strain for the linear axial and transverse sensors, respectively. It is seen that as the cycles progress, the resistance behavior finds a more repeatable trend. Similar to the strain softening and Mullins effects, the maximum drift in the resistance also occurs during the first cycle. It appears that the fundamental reasons for the changes in the mechanical and electrical behaviors during initial cycles are the same and associated with some permanent rearrangement, and in some cases, breakage of polymer chains within the polymer matrix, which causes an incomplete mechanical recovery and thus the exact initial percolative network of carbon nanotubes cannot be recreated. Once an equilibrium is reached for the strain cycling, a repeatable MWCNT network evolution is obtained.

**Figure 9:**
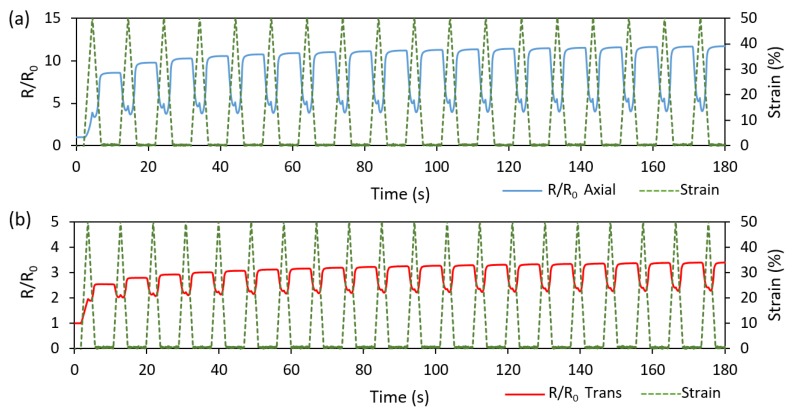
Cyclic relative resistance (R/R_0_) plots for (**a**) linear axial sensor (Figure 4a) and (**b**) linear transverse sensor (Figure 4b) loaded to 50% maximum strain.

Figure 9 demonstrates the cyclic piezoresistive behavior of both linear axial and linear transverse sensors over 20 cycles at 50% strain loading. For both sensor types, the resistance behavior showed a steady and consistent cyclic response after few initial cycles. Of interest is the inverse relation between the resistance and the strain in both sensor types. In the first cycle, the resistance increased during both the loading and unloading phases and a new higher level of resistance was established at the end of the first cycle. After the initial cycle, the sensors responded with a negative change in resistance, trending towards the resistance value that was established at the maximum strain of the first cycle. Upon the initial loading, the MWCNT network becomes disassociated, conductive paths are broken, new paths are potentially created, and the conductive trace finds a new conductivity equilibrium during unloading. However, on subsequent loadings, the MWCNT networks are rearranged during strain increase, reforming some of the previously disconnected networks [73,74].

This inverse type of resistance–strain relation was not observed in the monolithic sensors where the entire design volume was printed with the TPU/MWCNT (i.e., no platform/embedded trace pattern) [61]. This difference in the behavior is attributed to the stress state differences. The TPU/MWCNT conductive traces of the embedded sensors experienced a rather complex internal stress state during mechanical loading and unloading, as they were embedded and confined within the TPU platform with different mechanical properties. It thus appears that the driving force behind the piezoresistive response in the embedded sensors was two-fold. First, during the initial loading of virgin TPU/MWCNT nanocomposite, the response was driven by a decomposition of the conductive MWCNT networks. Further, during the first cycle strain unloading, the transverse compressive stresses exerted by the Poisson’s effect within the TPU platform decreased. This decrease in the stress allowed for a relaxation within the MWCNT network, further increasing the zero-strain resistance. Every strain loading after the first cycle re-applied the transverse compressive stresses, reestablishing the conductive network. Thus, an inverse relation between the strain and resistance was developed. It is also worth noting that the two-peak behavior usually observed in the cyclic resistance response of monolithic nanocomposite sensors [17,55,61] is minimized in the current embedded sensor designs. This feature makes the embedded design more appealing, as it would result in more reliable strain characterization. As demonstrated in the literature [54], the single-peak behavior and the cyclic repeatability in strain sensors with percolative characteristics can be enhanced by pre-straining of the sensor to register a more permanent pattern of destruction and reconstruction of percolative networks.

### 3.4. Biaxial Sensor Patterning

**Figure 10:**
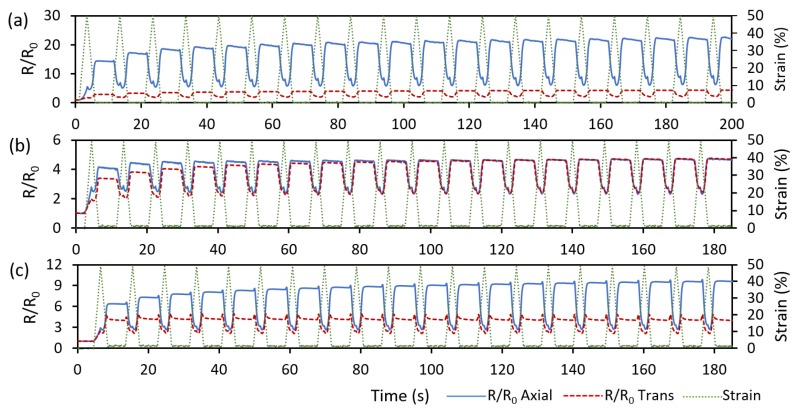
Cyclic piezoresistivity (R/R_0_) of biaxial sensors: (**a**) linear biaxial (Figure 4c); (**b**) switchback biaxial (Figure 4d); and (**c**) sawtooth biaxial (Figure 4e), loaded up to 50% maximum strain and unloaded to zero strain for 20 cycles.

One of the major benefits of FFF 3D printing is the ease-of-design when fabricating complex patterns. As a demonstration of the capabilities of FFF printed strain sensors, three sensor designs with different trace patterns were fabricated and compared: linear biaxial, switchback biaxial, and sawtooth biaxial (Figure 4c–e). Figure 10 shows the cyclic piezoresistivity of these three sensor variants when strained to a maximum of 50%.

The linear biaxial sensor demonstrates similar behavior to the results in Section 3.3 with the axial trace showing larger resistance sensitivity than the transverse trace by almost seven times (Figure 10a). As the conductive traces were deposited similar to a continuous wire, the strain response differs depending on whether the strain is being applied along the printed trace axially, as in the linear sensor’s axial trace, or radially, as in the linear sensor’s transverse trace. The linear sensor response exhibits a behavior that verifies this.

The switchback biaxial sensor demonstrates nearly identical responses in the axial and transverse directions. This can be attributed to the high degree of symmetry within the switchback sensor design. Another point of interest is the switchback sensor response being less sensitive than that of the linear sensor. The fact that the responses are similar in the axial and transverse directions of switchback design (Figure 10b) and differ markedly from that of the axial sensor response (Figure 10a) indicates that the more complex stress state at the curved locations of the switchback traces control the overall resistance. In other words, in the curved region of the traces, the MWCNT networks could not be reformed as effectively as they did in the straight axial traces.

Of significant interest is the sawtooth sensor response. The sawtooth sensor implements a trace design that is oriented with an angular offset from the axial and transverse directions. Therefore, unlike those of previous designs, the traces experience a complex stress state (a combination of axial and transverse stresses) that results in different strain levels. Compared to the applied transverse strains, the axial strains exhibit a greater effect on the sensor response, as seen in Figure 10c. Due to the off-axis orientation of the traces in the sawtooth design, the axial response experiences a decrease while the transverse response is enhanced. Therefore, by varying the sawtooth angle, different axial/transverse sensitivity combinations may be obtained. It is also noted that, as reported in [61], the electrical conductivity of FFF printed TPU/MWCNT nanocomposite is highly repeatable beyond its percolation threshold (>~2 wt %). In TPU/3 wt %MWCNT, the repeatability is very similar to that of compression-molded samples [61]. Therefore, as the piezoresistivity behavior is governed by the conductivity, these printed sensors are expected to be as reproducible as any other MWCNT-based sensors prepared using other fabrication methods such as compression molding.

The gauge factors of the sensors reported in this work vary between 1.5 and 3.0, which is well within the range of those reported for many nanocomposite-based strain sensors [21,75]. Sensors with gauge factors as high as 50 have also been reported but for much smaller strain ranges [76,77]. However, it is emphasized that the main goal of this work was enabling bidirectional and tunable strain sensing by integrating piezoresistive nanocomposite within the structure during manufacturing phase. If needed, higher gauge factors can be obtained for these sensors by further optimizing the carbon nanotube content of the nanocomposite and the sensor design.

### 3.5. Sensor Application

**Figure 11:**
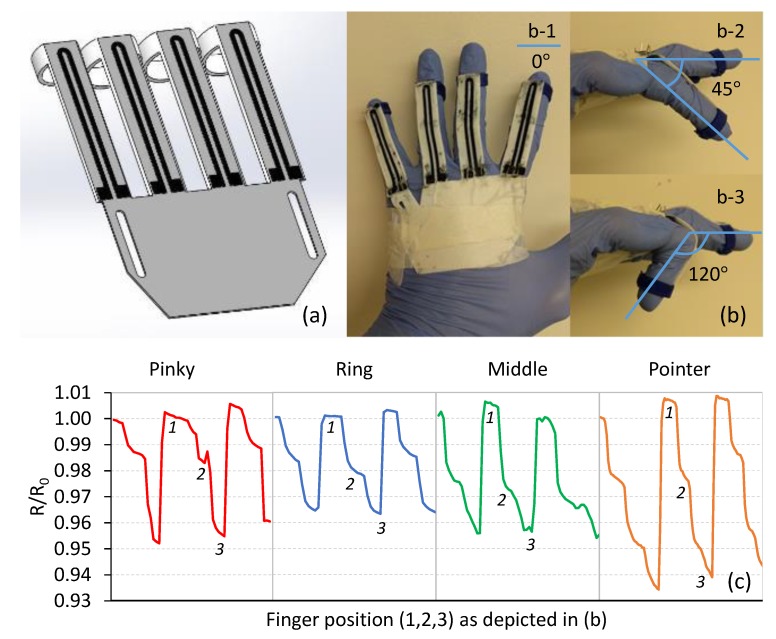
FFF 3D printed glove prototype for measuring finger flexure: (**a**) the CAD model; (**b**) the printed prototype worn on a hand shown in three different positions of b-1 (resting open, 0°), b-2 (45° bent) and b-3 (120° bent) during pointer finger flexure; and (**c**) the measured resistance response (R/R_0_) for three flexing cycles of each finger, where positions 1, 2 and 3 of the pointer finger are identified.

Within the field of highly flexible strain sensors, there is significant interest to develop wearable sensors capable of detecting flexure, pulse, and other functions [4,5,21,26]. As a demonstration of the capability of printed sensors, Figure 11 shows an FFF printed glove prototype using commercially available Ninjaflex filament for the sensor platform, the TPU/3 wt %MWCNT nanocomposite as the sensing traces, and commercial PLA filament for the finger rings. Figure 11a–c shows the CAD model of the glove, printed and mounted glove with finger sensors, and the responses to flexure. As seen in Figure 11c, the resistance response of the sensing elements was recorded when the fingers were bent to approximately 45° (Figure 11c-2) and 120° (Figure 11c-3) from a resting, open position (0°, Figure 11c-1). Each finger was flexed three times and relatively repeatable resistance responses were observed in all cases. It is noted that the sensitivity of the sensing elements embedded in the glove can be manipulated by the MWCNT content and the architecture of the printed traces.

## 4. Conclusions

With the rapid expansion of the 3D printing field, it is of great interest to demonstrate the strong potential for new sensor fabrication techniques. In this research, the capability of printing multi-material strain sensors through FFF printing was reported. It was shown that CNT-based TPU nanocomposites were capable of being printed in tandem with pure TPU for fabricating complex and unique geometries. The printed sensors demonstrated strong piezoresistive responses in both the axial and transverse directions over a wide range of strains. Good cyclic repeatability for the printed sensors was also reported for both electrical and mechanical performance, with steady-state behavior being reached relatively quickly (at ~4 cycles). Further, by properly patterning the sensor designs, it was possible to adjust the behavioral response. A wearable glove for measuring finger flexure was demonstrated as an application, where it was also shown that the TPU/CNT filament is capable of being printed with other commercially available filaments.

## Figures and Tables

**Table 1:** FFF 3D printing process parameters.

Parameter	Value
Print nozzle diameter (mm)	0.8
Nozzle temperature (°C)	220
Bed temperature (°C)	60
Layer resolution (mm)	0.2
Print infill (%)	100
Print speed (mm/s)	20
